# The use of digital storytelling of patients’ stories as an approach to translating knowledge: a scoping review

**DOI:** 10.1186/s40900-021-00305-x

**Published:** 2021-08-28

**Authors:** Elly Park, Mary Forhan, C. Allyson Jones

**Affiliations:** 1 Faculty of Rehabilitation Medicine, University of Alberta, Edmonton, AB T6G 2G4 Canada; 2 Department Occupational Therapy, Faculty of Rehabilitation Medicine, University of Alberta, Edmonton, AB T6G 2G4 Canada; 3 Department Physical Therapy, Faculty of Rehabilitation Medicine, University of Alberta, Edmonton, AB T6G 2G4 Canada

**Keywords:** Narrative medicine, Digital storytelling, Knowledge translation tools, Health research, Shared decision-making

## Abstract

**Background:**

A growing interest has centered on digital storytelling in health research, described as a multi-media presentation of a story using technology. The use of digital storytelling in knowledge translation (KT) is emerging as technology advances in healthcare to address the challenging tasks of disseminating and transferring knowledge to key stakeholders. We conducted a scoping review of the literature available on the use of patient digital storytelling as a tool in KT interventions.

**Methods:**

We followed by Arksey and O’Malley (Int J Soc Res Methodol 8(1):19–32, 2005), and Levac et al. (Implement Sci 5(1):69, 2010) recommended steps for scoping reviews. Search strategies were conducted for electronic databases (Medline, CINAHL, Web of Science, ProQuest dissertations and theses global, Clinicaltrials.gov and Psychinfo). The Preferred Reporting Items for Systematic Reviews and Meta-Analyses extension for scoping reviews (PRISMA-ScR) was used to report the review process.

**Results:**

Of 4656 citations retrieved, 114 full texts were reviewed, and twenty-one articles included in the review. Included studies were from nine countries and focused on an array of physical and mental health conditions. A broad range of interpretations of digital storytelling and a variety of KT interventions were identified. Digital storytelling was predominately defined as a story in multi-media form, presented as a video, for selective or public viewing and used as educational material for healthcare professionals, patients and families.

**Conclusion:**

Using digital storytelling as a tool in KT interventions can contribute to shared decision-making in healthcare and increase awareness in patients’ health related experiences. Concerns centered on the accuracy and reliability of some of the information available online and the impact of digital storytelling on knowledge action and implementation.

**Supplementary Information:**

The online version contains supplementary material available at 10.1186/s40900-021-00305-x.

## Plain English summary

Digital storytelling is a multi-media presentation of a story, often in the form of a narrated video. The use of digital storytelling of patient experiences with healthcare has gained attention in recent years, as a tool for sharing and understanding information among patients, caregivers, healthcare professionals and policy makers. A summary of the findings reported in studies looking at digital storytelling as a way of sharing information in healthcare is needed.

We searched literature that included the use of digital storytelling of patients’ healthcare experiences as a means of sharing and translating information, also referred to as knowledge translation or knowledge mobilization. There were 21 studies found from nine countries that used digital stories to look at experiences related to different physical and mental health conditions. A broad range of interpretations of digital storytelling and a variety of knowledge translation approaches were identified. The most common use of patients’ digital stories was educational material for healthcare professionals and other patients.

Using digital storytelling to translate knowledge can contribute to patients, caregivers, healthcare professionals and policy makers sharing the best available evidence when faced with making a health decision. Digital storytelling can help us understand patients’ health related experiences. Further work is needed to test the accuracy and reliability of some online information and how to best measure the impact of digital storytelling on knowledge translation activities.

**Supplementary Information:**

The online version contains supplementary material available at 10.1186/s40900-021-00305-x.

## Introduction

Sharing stories of healthcare experiences has become an approach to gain insight into a person’s perspective about their healthcare interactions, rather than focusing solely on an illness or condition [1]. Stories are a powerful tool for acquiring knowledge from patients about patients [2, 3]. Typically, patients tell their stories to others in oral or written form. The exchange of narratives between a patient and physician, described as narrative medicine, has been recognized as a model of care [2, 4]. As technology advances, stories are created and shared digitally, as a form of dissemination to a larger audience. Narrative medicine focuses on individual relationships between the healthcare provider and others, namely patients, other healthcare professionals, and society [4]. Patient narratives, as part of narrative medicine, have been used in clinical research and practice as a source of information, used as a tool for communication, engagement, persuasion, and health behavior change [5]. The focus and content of the narratives may differ, depending on the purpose within the clinical or research context [5, 6]. For example, the narrative may be an emotional account of receiving a diagnosis for a serious illness or could be a patient’s step by step account of daily insulin injections. Furthermore, the approach used to deliver stories will affect the reception from the audience [7]. For instance, using plain text as compared to a multi-media video with music and animations will impact the audience differently.

Digital storytelling has been defined as an arts-based multi-media presentation of a story, often in the form of a video [7]. It is also an accessible way of engaging patients within research, acknowledging the experiential knowledge they have to offer and creating connections with others including other patients, advocacy groups, caregivers, healthcare professionals and policy makers [3, 8–10]. Although limited research has employed patient digital storytelling in healthcare, it has been used as an educational tool for nursing students [11], healthcare intervention for patients with dementia [12], form of patient advocacy [9, 13], and method for communicating among women with HIV [14]. A systematic review protocol has outlined plans to examine methodological and ethical implications of digital storytelling in health research [10]. In spite of storytelling having a variety of uses not only at the micro level but also at the macro (policy) and meso (organizational) levels in healthcare. While no one theoretical model for digital storytelling has been embraced, Shaffer and colleagues [5] propose a Narrative Immersion Model that attempts to address the gaps in the literature by understanding the effects of health narratives on knowledge, attitudes and behavior. This theoretical model considers how narratives evoke different responses from other methods of sharing information. Here, we consider digital storytelling as a form of narrative. As such, we recognize that not all digital stories are equal, and we need to determine the purpose of using digital storytelling within individual studies to better understand the process and outcome of the stories [6]. Determining how or why digital storytelling is used within research is important to understand the outcomes and impact of this tool to minimize the knowledge-to-action gap.

The use of digital storytelling is a compelling approach for knowledge translation (KT) in healthcare because of how extensive, engaging and immediate the impact stories become when made accessible online [10, 15, 16]. Disseminating research findings to target audiences such as consumers and stakeholders is a complex task [17]. Successful KT activities requires planning and consideration of the knowledge providers and target audiences to ensure update of knowledge [18]. The use of digital stories to disseminate findings has not been explored. We conducted a scoping review to retrieve, review, and synthesize the current literature available [19] on digital storytelling as a tool to support different KT interventions in healthcare.

## Methods

Prior to conducting this scoping review, the review protocol was registered with Educational and Research Archives (ERA) (https://doi.org/10.7939/r3-ks11-ze31). The scoping review process followed the five stages proposed by Arksey and O’Malley [20] and Levac et al. [21]: (1) identifying the broad research question, (2) finding relevant studies, (3) study selection, (4) charting the data, and (5) collating, summarizing and reporting results. The optional sixth stage, consultation, was not included in this review. Recommendations outlined by Levac and colleagues [21] were closely followed throughout all stages of the review. To provide transparency of the process, we used the Preferred Reporting Items for Systematic Reviews and Meta-Analyses extension for scoping reviews (PRISMA-ScR) [22].

### Stage 1: identifying the research question

The population identified for this review included patients, caregivers, healthcare professionals and policy makers, the intervention was digital storytelling, and the outcome included KT tools. While several terms have been used to describe mobilizing knowledge into action, knowledge translation, knowledge transfer, implementation science and research utilization are frequently used terms [17, 23]. All study designs were included, while commentaries, editorials, conference abstracts, and letters or responses to letters were excluded. The specific research question was: How are patients’ stories using digital storytelling as a KT tool used to inform other patients, caregivers, healthcare professionals and policy makers about health-related topics? We wished to identify the key concepts and characteristics related to digital storytelling of patients’ stories for KT research in the available literature including grey literature and no restriction of study designs.

### Stage 2: identifying relevant studies

A library health information specialist developed and implemented the search for six electronic databases (MEDLINE, CINAHL, Web of Science, ProQuest dissertations and theses global, Clinicaltrials.gov, and PsycINFO). We narrowed the search to include literature published within the past 10 years (2009–2019) to May 16, 2019 based on technological advancements and secular trends. Digital storytelling has only recently been identified as a tool in healthcare [7, 8], and therefore, this time frame would sufficiently capture the most relevant information. The search terms included *digital storytelling, personal narratives, multimedia, health research, healthcare, patient experience, patient engagement, knowledge dissemination and translation,* as well as related terms with truncations [See Additional file 1 for detailed search strategy]. Citations were restricted to English only. Restricting the search to English studies was based on findings from systematic research evidence that reported no empirical evidence of bias seen if papers were written in languages other than English [24]. Grey literature from non-peer reviewed sources were searched specifically through the database ProQuest dissertations and theses global and included on a case-by-case basis, based on relevance to the research question.

Abstracts had to include digital storytelling focusing on health-related patient experiences. The inclusion criteria were based on research question and aim to identify and review research of digital storytelling for KT as defined by the Canadian Institutes of Health Research (CIHR): “a dynamic and iterative process that includes synthesis, dissemination, exchange and ethically-sound application of knowledge to improve the health, provide more effective health services and products and strengthen the health care system” [25]. Knowledge translation strategies were identified and categorized as: educational materials, knowledge exchange, mass media, and community outreach [18, 26, 27]. Specifically, we identified current literature which used digital storytelling as a KT tool targeting patients, caregivers, healthcare professionals and policy makers [11, 28, 29]. To ensure a comprehensive review, we included all studies that had a KT component using digital stories regardless of whether it was explicitly stated as part of the methodology.

### Stage 3: study selection

Citations were uploaded to Covidence [30], which is web-based software platform that streamlines the production of systematic reviews. Duplicate records were identified and removed at this stage. Titles and abstracts were independently reviewed by 2 reviewers (EP, SR) with reported “strong” (kappa = 0.82) inter-rater reliability agreement [31, 32]. Disagreements in study selection were resolved by discussion between the 2 reviewers, and when necessary, through third-party adjudication (MF, CAJ) if the reviewers did not arrive at consensus. Full text screening of included abstracts and titles was then completed by the same 2 reviewers.

### Stage 4: charting the data

Data were extracted from the first five studies independently by both reviewers using a standardized extraction form. The reviewers discussed their findings and found a high level of consistency in the data extracted from the studies. After explicitly clarifying each of the data extraction points, one reviewer (EP) then completed data extraction for the remaining sixteen studies. Data based on the population, digital storytelling, KT intervention and study characteristics were extracted and documented in a Microsoft excel spreadsheet.

### Stage 5: collating, summarizing and reporting results

After data extraction, the results were collated and summarized to include study characteristics based on the research question. The descriptive table presented the year of publication and country of origin, the purpose of the study, as well as the study design, target population, the rationale for using digital storytelling and the type of KT intervention. Key findings were outlined in the reported results and then synthesized and mapped based on the type of digital storytelling used, and the type of KT intervention.

## Results

### Study characteristics

**Fig. 1:**
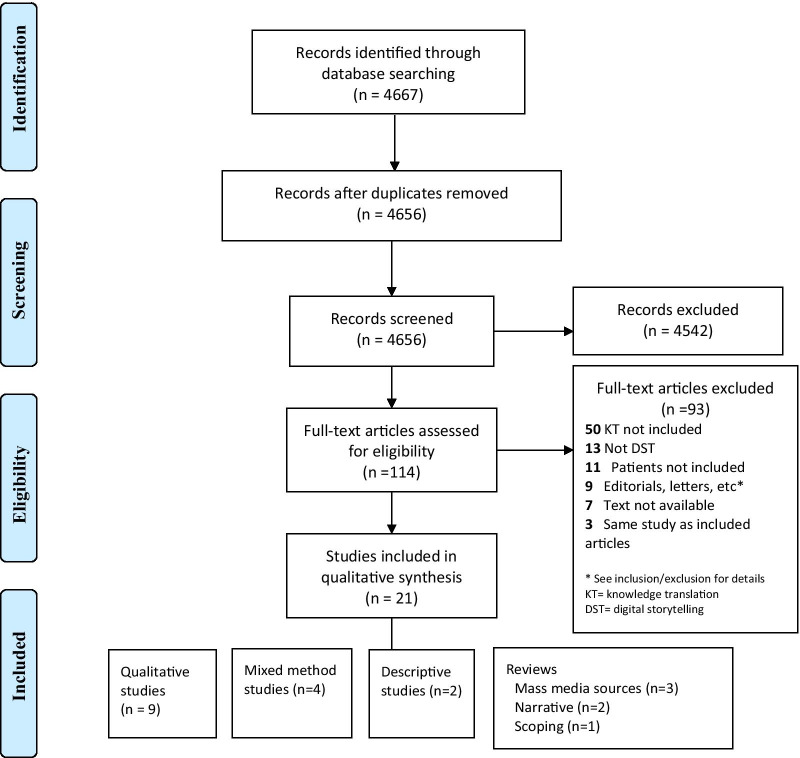
PRISMA flow chart. *Source*: Moher D, Liberati A, Tetzlaff J, Altman DG, The PRISMA Group (2009). *P*referred *R*eporting *I*tems for *S*ystematic Reviews and *M*eta-*A*nalyses: The PRISMA Statement. PLoS Med 6(7): e1000097. https://doi.org/10.1371/journal.pmed1000097

Of the 4667 citations retrieved, 11 duplicates were removed, and the remaining 4656 citations were reviewed for eligibility. Based on the criteria outlined in Table 1, twenty-one studies were included in the review (Fig. 1). The majority of excluded studies (n = 50) did not include a KT component. Several studies were excluded because they were not research studies (n = 9), did not include digital storytelling (n = 13), or did not include patients (n = 8). Citations that did not have an available full text were excluded because they were conference abstracts (n = 5) or the authors did not respond to our full text request (n = 2).

**Table 1:** Inclusion criteria

Research component	Criteria
Study design	All study designs were included/commentaries, editorials, conference abstracts, letters or responses were excluded
Intervention	Digital storytelling of patients’ experiences is used as part of KT in health research
Focus of study	Healthcare related
Outcomes	Knowledge translation to other patients, caregivers, healthcare professionals, policy makers

Studies were from nine countries including seven from USA [33–39], five from Canada [13, 40–43], three from the United Kingdom [9, 15, 44], and one each from Australia [45], Dubai [46], Sweden [16], Italy [47], Germany [48] and Taiwan [49]. Nine studies were qualitative methods [15, 16, 34, 36–38, 41, 43, 49], four used mixed methods [35, 39, 42, 48], three were media reviews [40, 46, 47], two narrative reviews [9, 13], two descriptive studies [33, 44] and one was a scoping review [45]. Three studies were published graduate dissertations [9, 37, 38], with one being a narrative review [9] and two using qualitative methods [37, 38].

Study populations included children and youth [41, 42, 47] adults [15, 16, 34–38, 40, 48, 49], and older adults [43] with six studies not specifying the target population [9, 13, 33, 44–46]. Health related topics included health promotion [37, 38], dementia [43], chronic pain [42], mental health [45], infertility [40], inflammatory bowel disorder [36], post-traumatic stress disorder [16], chronic obstructive pulmonary disorder [33], diabetes [34, 46], and cancer [15, 35, 41, 47–49]. Four studies did not focus on a specific health condition [9, 13, 39, 44] but referred broadly to patients in healthcare settings (Table 2).

**Table 2:** Study characteristics of included studies by digital storytelling method

Author/year/country	Study aim/purpose	Study design	Rationale for digital storytelling method	Target population	Type of KT	Findings
*Audio/visual presentations (video format)*						
Adams et al./2015/UK	To explore the long-term impact of filmed patient narratives (“patient films”) as sources of meaningful and reliable knowledge	Qualitative—ethnographic evaluation	To provide healthcare providers with stories of patient experience	Healthcare professionals	Educational material	Presenting patient stories encouraged reflection and reflexive learning The “patient films” were reported as powerfully persuasive This knowledge was associated with intimacy, “real communication” Digital stories were contrasted with the disinterested and impersonal expertise of statistics
Benson/2012/USA	To evaluate how digital stories contributed to goals of policy change, engaging community members and increasing understanding of health disparities	Qualitative—community forums, written surveys, structured observations (thesis)	To engage community to increase awareness, and lead to policy change	Patients, caregivers and policy makers	Educational material, community outreach to influence policy	Digital stories provided a fresh perspective of health disparities in an authentic way Using digital stories in community forum facilitated policy changes in all four areas and promoted community engagement
Canning and Phinney/2015/Canada	To explore the use of data documentary film for accessing the subjective experiences of the participants, and for knowledge translation	Qualitative—visual narrative	To inform healthcare professionals and policy makers in a personal way about living with dementia	Healthcare professionals, policy makers	Educational material, community outreach to influence policy	Using documentary data to hear voices of people with dementia brought the viewer into the experience of participants The film elicited strong emotions by viewers in a powerful and engaging way In dementia research, using documentary films as a KT tool is effective for bringing different community members together
Clerici et al./2012/Italy	To investigate the availability and type of video content on YouTube in relation to rhabdomyosarcoma and soft-tissue sarcoma in children	Media review—cross-sectional methods	To share strategies for coping and other available resources	Patients, caregivers and healthcare professionals	Mass media, knowledge exchange	Video-sharing sites such as blogs and social media made it easier for patients to describe their impressions and experiences of the disease Their experiences helped other patients devise strategies for coping with the disease Sharing online provided patients with support and opportunities for exchanging information and resources
De Vecchi et al./2016/Australia	To identify how and in what areas digital storytelling has been used in mental health	Scoping review	To enhance relationships between patients and healthcare professionals during the recovery process	Patients, caregivers and healthcare professionals	Educational material, knowledge exchange	May enable consumers, carers and healthcare professionals to work together to learn about, understand and empathize with each other’ s lived experience Has implications for the development of recovery oriented mental health services Lack of uptake of this method in research in mental health services may be a missed opportunity
Hardy/2016/UK	To document the development of digital storytelling in healthcare and to examine, the contribution made via the Patient Voices Programme, to the wider genre of digital storytelling	Narrative summary of multiple studies—qualitative methods (dissertation)	To focus on health education, advocacy, and quality improvement	Patients, caregivers, healthcare professionals and policy makers	Mass media, educational material	Patient Voices Programme has developed over 1000 digital stories in health Patient stories have been used to understand experiences to balance statistical evidence about health experiences Provided evidence that digital storytelling can be adapted for use in different contexts Used a solid ethical foundation to protect those sharing stories
Kelly-Hedrick et al./2018/Canada	To (1) describe the video content of the most highly viewed fertility-related YouTube videos and (2) identify video characteristics that relate to viewer favourability (eg, video likes, shares)	Media review—content analysis	To disseminate information about fertility to other patients	Patients and partners	Mass media, knowledge exchange	Reliance on YouTube for infertility information may foster unrealistic expectations regarding the success rates of treatment, which may influence treatment decisions As fertility patients frequently access the internet for information, research is needed to establish whether YouTube content can affect the perceptions of infertility treatment among fertility patients
Laing et al./2017/Canada	To understand the effect of watching digital stories on healthcare professionals made by (past and present) pediatric and young adult oncology patients, including usefulness, and impact on practice	Qualitative—hermeneutics	To share digital stories with healthcare professionals, as a way to inform and enhance understanding of young adult cancer related experiences	Healthcare professionals	Educational material	Reflective digital storytelling powerfully assisted practitioners to understand patients’ experiences Both the creation and viewing of digital stories can lead to new insight, understanding, and more generative approaches to care Viewing digital stories were about understanding an experience versus simply explaining it, and therefore may be at the heart of patient engagement and health care delivery in general Digital stories have practical implications in health care systems that struggle to do more with fewer resources
Lal et al./2015/Canada	To introduce digital storytelling and to propose how it can be applied in occupational therapy	Narrative review	To review potential uses of digital storytelling in occupational therapy	Healthcare professionals	Educational material	Promising for application within the field of occupational therapy—as a useful tool for therapists, educators, and researchers Digital stories can facilitate the dissemination of research findings in accessible and engaging ways, conveying key messages to promote the quality of care
Shapiro et al./2009/USA	To learn about patients' lives through making films collaboratively with medical students and patients	Mixed methods—interviews, quantitative evaluations	To understand patients using an engaging and collaborative method	Healthcare professionals	Educational material, knowledge exchange	Students reported that the project affected what they planned to cover in clinic visits, increased their plans to involve patients in care, enhanced their appreciation for patient-centered care, improved their knowledge of community resources, improved their understanding of allied health professionals’ roles, and taught them about patients’ innovative adaptations
*Multiple resources with embedded stories*						
Engler et al./2016/Germany	To investigate how a website that presents narratives of cancer patients is used by other cancer patients, and what users expect and learn from such a website	Mixed methods—log file and survey data analyses, thematic analysis of focus groups	Website with patient stories and videos to enhance knowledge of other patients	Patients and caregivers	Educational material, knowledge exchange	A valuable and beneficial resource that provided a wide range of diverse experiences Participants used the website to find people with similar characteristics They reported that the stories helped them to reflect on their own situation and the narratives provided hope and gave them confidence
Marir et al./2014/Dubai	To extract and store knowledge about diabetes diagnosis, diet, lifestyle, medicine, and activities from social networks, patient stories and other text document sources	Media review—text mining	Database of patient experiences to provide information to healthcare professionals about patients with diabetes	Healthcare professionals	Educational material	Knowledge repository can be useful for diabetes based on knowledge discovered from applying text mining to raw textual data collected from social networks MEDLINE medical publication records is feasible These data were stories of people telling their accounts of diagnosis, i.e. how they found out about it, their lifestyle before, as well as the certain events and actions that led to the realization of the disease
Stellefson et al./2018/USA	This study reviews a free, publicly available online community and support network hosted by the COPD Foundation known as COPD360social	Descriptive summary	Social network for communication and knowledge exchange regarding COPD	Patients, caregivers and healthcare professionals	Mass media, educational material, knowledge exchange, community outreach	COPD360social is an innovative social networking initiative that has improved how patients, caregivers, and practitioners communicate about COPD related research, education, and advocacy It provided an interactive network that fosters patient education and community engagement For health education/promotion practitioners, COPD360social served as a reputable, inclusive network for COPD-specific emotional, instrumental, and informational support
Ziebland et al./2015/USA	The DIPEx International project (http://www.dipexinternational.org) is a collaboration of qualitative health researchers which present analyses and hundreds of video and audio interview extracts	Descriptive summary	Videos of patient experiences on a website to be shared as a resource with other countries	Patients, caregivers and healthcare professionals	Mass media, educational material	The Internet presents opportunities to transform the experience of chronic pain and other health conditions by drawing on people’s willingness to share their experiences online One implication is locally led, culturally appropriate projects to produce e-health resources in developing countries and from marginalized communities in developed countries
*Interactive platform/multiple contributors*						
Chiu and Hsieh/2012/Taiwan	To understand why cancer patients write on the internet, including their blogs and support group sites and how writing and reading other patients’ messages impact their cancer experiences	Qualitative—focus groups, grounded qualitative analysis	Blogs and online forums to exchange information with other patients and caregivers	Patients and caregivers	Mass media, knowledge exchange	Health professionals could help or encourage newly diagnosed cancer patients to write and interact with other patients on the internet to assist them in coping with their illness and acquire social support Participants reported that sharing stories was therapeutic, meaningful, and empowering
Frohlich/2019/USA	To determine how leaders of online Inflammatory bowel disease communities perceive their responsibility for disseminating health information to other people with inflammatory bowel disease	Qualitative—digital ethnography	Online chat communities (forums) for information dissemination and exchange	Patients and healthcare professionals	Mass media, knowledge exchange	Personal experiences as another kind of health information was sometimes more helpful than technical information Community members noticed there was little interaction between medical authority and patient authority members Online communities were used to gain information about how inflammatory bowel disease affects one’s daily life, something medical professionals did not offer
Reid et al./2017/Canada	To work with children attending the pediatric chronic pain clinic and their parents to develop, refine, and evaluate the usability of narrative-based e-book for pediatric chronic pain	Mixed methods—interviews, feedback, usability evaluation	An e-book of a patient with pediatric chronic pain, based on patient experiences to be shared with parents as a KT tool	Caregivers	Educational material	Parents preferred receiving health information in a narrative form rather than the standard information-based format Parents preferred the e-book over standard ways of receiving health information, and they were likely to recommend the e-book to others There was an increase in knowledge about the topic after reading the e-book
Salzmann-Erikson and Hicdurmaz/2017/Sweden	To describe how individuals suffering from post-traumatic stress use social media to communicate authentic narratives of their life conditions	Qualitative—descriptive, netnographic (online qualitative research)	Online communities (forums) for communication and knowledge exchange among patients with PTSD	Patients and caregivers	Educational material, knowledge exchange	Computer mediated communication provided PTSD sufferers a way to reveal themselves, to experience the universality of the problem, to receive support and help from other people, and to fight against stigma
*Workshop based digital stories*						
Cueva et al./2016/USA	To understand (a) how creating a cancer-related digital story affects Alaskan community health aid /professionals’ (CHA/Ps) cancer knowledge, attitudes, and health behaviors, and (b) how the CHA/Ps used digital stories as health communication tools	Mixed methods—group teleconference, end of course evaluation, surveys	Workshop based patient and caregiver experiences videos disseminated in Alaskan communities as a KT tool	Patients and healthcare professionals	Educational material, knowledge exchange, community outreach	Cultural relevance, brevity, reproducibility, and flexibility of digital stories may be widely translated into diverse settings and locales, increasing their impact potential Indigenous digital stories have the potential to open cancer conversations and impact cancer prevention and detection behaviors to change the story of cancer among Indigenous people
Halter/2015/USA	To learn how the use of digital stories can advance the growing community partnership created in the Yakima Valley and improve and further promote health equity	Qualitative—community Based Participatory Research, interviews and focus groups (dissertation)	Workshops to capture patient experience to advance a community partnership with local health organization	Patients, caregivers and healthcare professionals	Educational materials, community outreach	For storytellers, it provided a healing outlet to critically reflect on a difficult experience and find support within one’s own community For viewers, digital stories were far more impactful than other forms of health education materials that may not be culturally appropriate or accessible to the members of this population The engagement process of building capacity and collaborating within a community was an important way for community organizations to foster relationships within the community
Njeru et al./2015/USA	To report on the process and outcomes of the participatory development of a diabetes storytelling intervention as a KT tool	Qualitative—community Based Participatory Research, focus groups	Workshop based digital stories of patient experience for people with diabetes to be shared with the Somali and Latino communities	Patients and caregivers	Educational material, Community outreach	The participatory processes of developing a digital diabetes storytelling intervention to improve type II diabetes management with Somali and Latino participants may be useful for other partnerships seeking to develop homegrown interventions to address local health concerns with and for community partners

### Digital storytelling terminology

While the interpretation of what is digital storytelling varied within these studies, the majority defined digital storytelling as a story in multi-media form, presented as a video, for selective or public viewing [9, 13, 15, 34, 35, 37–41, 47]. Other studies referred to digital storytelling as an exchange of information and communication using technology, such as through online patient forums and websites [33, 36, 44, 48, 49]. The target audience for the digital stories varied to include patients, caregivers, healthcare professionals and policy makers. For this review, all the stories were based on the patients’ experiences from the patients’ perspectives, although in some cases they were created in collaboration with healthcare professionals and researchers [9, 15, 35, 39, 42, 43].

Several types of digital storytelling were found including: (a) personal multi-media patient stories with a KT component such as digital stories created with researchers and patients in a workshop [9, 13, 15, 34, 35, 37–39, 41], (b) multiple resources with embedded stories such as health-related websites with patient stories [9, 33, 44, 46, 48], (c) interactive storytelling such as blog postings within online communities to share stories and information [16, 36, 45, 49], (d) audio/visual presentations such as YouTube videos or published documentaries with recordings of patient stories [40, 43, 47], and (e) digital educational resource for parents based on collective patient experiences such as an e-book [42].

The purpose of the stories also varied within the different forms of digital storytelling; yet, the primary reason for using digital storytelling in several studies was to inform and exchange knowledge [15, 33, 35–37, 39, 41–44, 46, 48]. Some stories served multiple purposes and, in several cases, digital stories were effective in engaging, comforting and informing the target audiences [16, 36, 40, 49]. In studies where health promotion or policy change was an objective, the digital stories served to engage and persuade the general public including policy makers and other stakeholders [34, 37, 38]. Digital storytelling was used in an array of KT strategies, predominantly for educational purposes.

### Knowledge translation

Most studies used several KT strategies, except for six studies that used digital stories solely for relaying professional/patient education material [13, 15, 39, 41, 46]. Overall, digital storytelling was mainly used as a KT tool for educational purposes (n = 17) to inform healthcare professionals, patients and caregivers [9, 13, 15, 16, 33–35, 37–39, 41–46, 48]. A few studies, focused on health promotion to educate the general public [34, 37, 38]. In addition, KT strategies included information dissemination through mass media (n = 8) where social media and websites were used as platforms for KT of the digital stories [33, 36, 40, 44, 45, 47, 49]. Ten interactive sites provided opportunities for knowledge exchange, primarily for patients [16, 33, 35, 36, 40, 44, 45, 47–49]; however, five studies of these studies had overlap with mass media which permitted public access [36, 40, 44, 47, 49]. Community outreach (n = 6) focused on health behavior change, health promotion, and policy changes [33–35, 37, 38, 43].

Several studies discussed the power of patients’ digital stories to improve knowledge acquisition by being more accessible and engaging [15, 41, 43, 44]. Digital stories were easily retrieved by other patients, caregivers, healthcare professionals and the public, to complement or enhance the technical data presented as statistics [37, 44, 47]. The digital stories offer a deeper understanding of the patients’ healthcare experiences, including interactions with healthcare professionals and receiving assessments, education and treatment [45]. The knowledge included in digital stories were often compelling and persuasive to engage the audience and promote information retention [15, 34, 41–43]. Through digital storytelling, patients can provide perspectives to key stakeholders that may not have been otherwise, considered [15, 41].

**Fig. 2:**
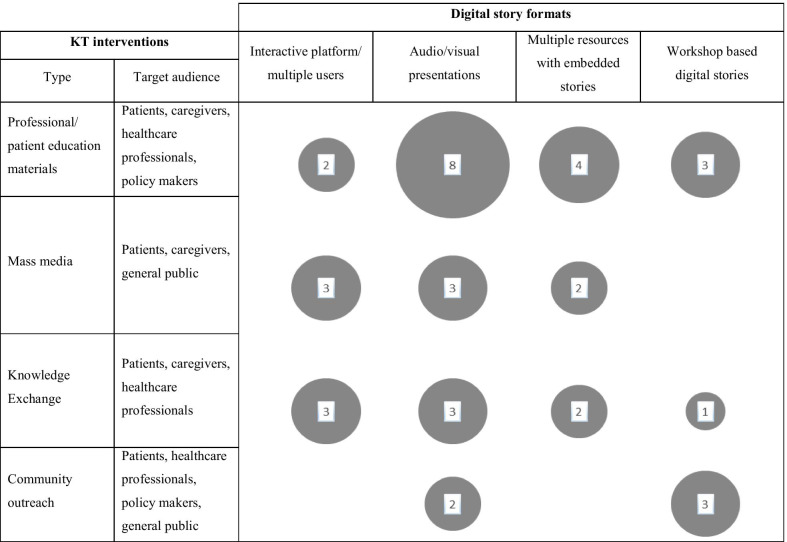
Evidence map of the number of studies for type of knowledge translation and type of digital storytelling. (Number of studies is represented by the size of circle and the number seen in circle)

The digital storytelling types and KT interventions were mapped (see Fig. 2) to depict the different forms of digital storytelling used with different KT strategies. A majority of studies used digital stories as *educational material* for healthcare professionals, patients, caregivers and policy makers. The aims of these studies included training healthcare professionals [9, 13, 15, 35, 39, 41, 43, 45], analyzing the use of available social media and resources as educational tools for patients [16, 34, 45, 48], creating a database for healthcare researchers and patients to access [33, 44, 46], and using digital stories for public health education [34, 37, 38]. *Mass media* was used as a form of dissemination of information to a public audience, at times coinciding with digital stories as an educational resource. Patients have seized the opportunity to access this readily available information, and used social media platforms to interact with other patients and healthcare providers [36, 40, 49]. The use of interactive digital platforms for *knowledge exchange* may indicate a growing trend of acquiring knowledge through virtual communities, locally and internationally. These studies highlight patients’ desire to connect with other people who have similar experiences, appreciating the experiential knowledge they may offer [16, 33, 36, 49]. Another KT strategy, *community outreach* was used to examine health and social disparities, drawing on digital storytelling to bring communities together to promote awareness and to influence policy [34, 35, 37, 38].

## Discussion

Findings from this review indicate that digital storytelling of patients’ health experiences is an emerging strategy for translating knowledge in healthcare. A large portion of the studies using digital storytelling for KT were published in the last five years (n = 16) which reflects the recent development of this approach for KT purposes. Digital storytelling used several different KT strategies for health research to enhance accessibility, education, and community building. Various forms of digital storytelling were discussed in our findings including an interactive exchange of stories, published videos, and patient stories embedded in websites about health conditions. In most cases, digital stories were part of educational resources for other patients, caregivers and healthcare professionals. Using a digital storytelling approach received a positive reaction by the target audiences as an engaging and powerful way of providing relevant and meaningful information.

Shared decision-making (SDM) and patient engagement have become an important elements of KT, in Canadian health research [50] and internationally [51]. In the past ten years, patients have been increasingly involved in KT research, as part of the CIHR initiative Strategies in Patient Oriented Research (SPOR), focused on patient involvement in health research [50]. An international organization, the International Patient Decision Aid Standards (IPDAS), consists of patients, clinicians, researchers, and policy makers, who have created and continue to update standards to ensure quality and effectiveness in the development and use of patient decision aids [51]. Patients want to be informed and aware of options available as well as be in a position to make choices based on reliable information [52, 53]. Patients may relay their stories of experience to contribute to the SDM process by providing other patients, caregivers, healthcare professionals and poly makers firsthand experiential knowledge. By sharing personal health experiences and hearing from other patients, the digital stories may help patients feel more confident to advocate for themselves. Health researchers recognize the need to offer opportunities for patients to be involved at various stages of research, to inform and support the decision making process [50, 51].

Healthcare professionals viewed digital storytelling as a viable KT tool in the form of interactive, knowledge exchange platforms for patients, caregivers and healthcare professionals to share knowledge and expertise. One concern of media-based digital storytelling is the quality or accuracy of information which is not typically monitored or substantiated by experts. Healthcare professionals have expressed concerns that patients do not have the expertise to provide reliable, evidence-based medical advice, creating a ripe opportunity to collaborate with patients and develop resources that are accessible and relatable. In spite of these concerns, patients appreciated the opportunity to share their stories with other patients and caregivers to provide support, insights and hope. Digital storytelling of patients’ experiences must include the voices of the patients while offering accurate and credible information. Drawing on resources from SPOR [50] and IPDAS [51] may provide patients and healthcare professionals with a guide to support collaboration and ensure reliability.

Our findings highlight the benefits of using digital storytelling in KT research including the use of the internet to improve access and engagement by patients and caregivers. Healthcare professionals were provided opportunities to gain a deeper understanding of their patients beyond their health conditions or diagnoses. For culturally diverse communities where health promotion and public health education may be more challenging, digital storytelling is a meaningful way to communicate and connect with community members. Inasmuch as no evidence-based framework for digital storytelling is endorsed within the literature, this review provided an overview of the extensive ways digital storytelling has been used. We also offered an outline of the different purposes and outcomes in the studies using digital storytelling as a KT tool, based on population and research questions. Synthesizing and mapping the evidence by type of digital storytelling, target population, and type of KT interventions offers a visual representation, summarizing the analysis in our scoping review.

Although methodological inclusivity allowed us to review diverse studies, limitations of this review included the inability to access some potentially relevant studies. Seven citations were excluded because the full texts were not available. The quality of the research of articles included was not assessed because of various types of research designs.

Measuring the impact of digital stories as a KT tool has not been investigated and is a gap in the available research. In many studies, the digital stories were publicly available online, but the impact on knowledge users was not evaluated. Those who accessed the digital stories, mainly patients and caregivers, reported that watching and hearing patient experiences was more appealing as compared to statistical or technical presentations. Evaluating the impact of digital storytelling in terms of knowledge acquisition, stakeholders’ understanding of patients’ experiences, and changes in behavior could provide rationale for supporting the use of digital storytelling with KT interventions in healthcare.

## Implications

The use of technology, namely the media-based digital storytelling, allows patients and other stakeholders to access relevant information, but may lead to inaccurate or unsubstantiated material being distributed. Ensuring that appropriate healthcare professionals are aware of these resources and involved in knowledge dissemination could ensure the messages are corroborated and verified by trained specialists, while preserving the message being conveyed by patients.

This scoping review provides a synthesis of the digital storytelling literature used as a tool for KT interventions. Our findings highlight the benefits of using digital storytelling, including audience engagement and information retention, as well as facilitating patient involvement in the KT process. To maximize the potential of digital storytelling in KT activities, development of a framework would be helpful to guide the use of digital platforms and ensure consistency. Furthermore, it can support researchers and clinicians in making decisions of what types of digital storytelling to focus on, based on their desired outcomes and aims for KT.

## Conclusions

The use of digital storytelling has emerged as a KT strategy in healthcare especially over the past five years; however, concerns with the accuracy and reliability of some of the available online information warrant further research. Evaluating the impact of digital stories in terms of knowledge transfer and implementation will provide valuable information as to the effectiveness of digital storytelling, particularly as different forms of digital storytelling emerge. There has been limited research on the extent of this impact, and how it may lead to changes in beliefs and/or behaviors warrants further investigation. Developing a framework to facilitate future research in this area would help patients, caregivers, health researchers, healthcare professionals and policy makers understand the types of digital storytelling and determine the most suitable option based on their KT goals.

## Supplementary Information

**Additional file 1.** Medline Search Terms.

## Data Availability

Not applicable.

## Abbreviations

CHA/Ps: Community health aid /professionals’
CIHR: Canadian Institutes of Health Research
CINAHL: The Cumulative Index of Nursing and Allied Health Literature
Embase: Bibliographic database for biomedical research
ERA: Educational and Research Archives
IPDAS: International Patient Decision Aid Standards
KT: Knowledge translation
MEDLINE: Medical Literature Analysis and Retrieval System
PRISMA-ScR: Preferred Reporting Items for Systematic Reviews and Meta-Analyses extension for scoping reviews
PsycINFO: Psychological Information Database
SDM: Shared decision-making
SPOR: Strategies in patient oriented research
