# Supplementary material for: The use of digital storytelling of patients’ stories as an approach to translating knowledge: a scoping review
Source: Res Involv Engagem. 2021 Aug 28;7:58. doi: 10.1186/s40900-021-00305-x (PMC8403386; doi:10.1186/s40900-021-00305-x)
Supplement: Supplementary file 1 — Additional file 1. Medline Search Terms. [file 40900_2021_305_MOESM1_ESM.docx]

**Supplement File 1**. Medline Search Terms

Database: Ovid MEDLINE(R) and Epub Ahead of Print, In-Process & Other Non-Indexed Citations,

1 research/

1. health services research/
2. ((health or healthcare) adj2 (education$ or research$ or service?)).mp.
3. qualitative research$.tw,kf.
4. education/mt
5. exp education, professional/mt
6. health education/mt
7. patient education as topic/
8. information dissemination/
9. "diffusion of innovation"/
10. ((client? or consumer? or doctor? or "health care profession$" or nurs$ or patient? or peer? or pharmacist? or physician? or practitioner? or researcher?) adj3 (educat$ or experien$ or inform$ or instruct$ or knowledg$ or program$ or teach$ or tool?)).tw,kf.
11. (knowledge adj2 (disseminat$ or transfer$ or translat$ or support$ or uptake)).tw,kf.
12. ((experienc$ or experiential) adj2 learning).tw,kf.
13. (patient? adj2 (engage$ or perspective?)).tw,kf. (16350)
14. or/1-14
15. narrative therapy/
16. narration/
17. personal narratives as topic/
18. ((narration? or narrative?) adj3 (stories or story or storytell$)).tw,kf.
19. narrative therap$.tw,kf.
20. (patient$ adj2 (diaries or diary or narration? or narrative? or stories or storytell$)).tw,kf.
21. (audio$ adj4 (stories or story or storytell$)).tw,kf.
22. (audio$ adj4 (clip? or diaries or diary or narrative?)).tw,kf.
23. (digital$ adj4 (stories or story or storytell$)).tw,kf.
24. (digital$ adj4 (diaries or diary or narrative?)).tw,kf.
25. (multimedia$ adj4 (stories or story or storytell$)).tw,kf.
26. (multimedia$ adj4 (diaries or diary or narrative?)).tw,kf.
27. (video$ adj4 (stories or story or storytell$)).tw,kf.
28. (video$ adj4 (diaries or diary or narrative?)).tw,kf. (216)
29. or/16-29

31 15 and 30

32 limit 31 to yr="2009 - 2019"

33 limit 32 to english language
